# Predictive factors for successful vagus nerve stimulation in patients with refractory epilepsy: real-life insights from a multicenter study

**DOI:** 10.3389/fnins.2023.1210221

**Published:** 2023-07-27

**Authors:** Henrique Jannuzzelli Pires do Prado, Lécio Figueira Pinto, Daniela Fontes Bezerra, Luciano de Paola, Francisco Arruda, Andrea Julião de Oliveira, Tayla Taynan Romão, Vanessa Cristina Colares Lessa, Jonadab dos Santos Silva, Isabella D’Andrea-Meira

**Affiliations:** ^1^Department of Epilepsy, Instituto Estadual do Cérebro Paulo Niemeyer, Rio de Janeiro, Brazil; ^2^Postgraduate Program in Neurology/Neurosciences, Universidade Federal Fluminense, Niterói, Brazil; ^3^Department of Epilepsy, Hospital das Clínicas da Faculdade de Medicina USP, São Paulo, Brazil; ^4^Department of Epilepsy, Faculdade de Medicina do ABC, São Paulo, Brazil; ^5^Department of Epilepsy, Universidade Federal do Paraná, Curitiba, Brazil; ^6^Department of Epilepsy, Instituto de Neurologia de Goiânia, Goiânia, Brazil; ^7^Department of Epilepsy, Hospital Felicio Rocho, Belo Horizonte, Brazil; ^8^Department of Neurology, Icahn School of Medicine at Mount Sinai, New York, NY, United States

**Keywords:** epilepsy, neuromodulation, drug-resistant epilepsy, vagus nerve stimulation, prediction response

## Abstract

**Introduction:**

Vagus nerve stimulation (VNS) therapy is an established treatment for patients with drug-resistant epilepsy that reduces seizure frequency by at least 50% in approximately half of patients; however, the characteristics of the patients with the best response have not yet been identified. Thus, it is important to identify the profile of patients who would have the best response to guide early indications and better patient selection.

**Methods:**

This retrospective study evaluated vagus nerve stimulation (VNS) as an adjuvant therapy for patients with drug-resistant epilepsy from six epilepsy centers in Brazil. Data from 192 patients aged 2–66 years were analyzed, and all patients received at least 6 months of therapy to be included.

**Results:**

Included patients were aged 2–66 years (25.6 ± 14.3), 105 (54.7%) males and 87 (45.8%) females. Median follow-up interval was 5 years (range, 2005—2018). Overall, the response rate (≥50% seizure reduction) after VNS implantation was 65.6% (126/192 patients). Most patients had 50–90% seizure reduction (60.9%) and nine patients became seizure-free. There were no serious complications associated with VNS implantation. The rate of a ≥ 50% seizure reduction response was significantly higher in patients with no history of neurosurgery. The presence of focal without generalized seizures and focal discharges on interictal EEG was associated with better response. Overall, etiological predictors of a better VNS response profile were tumors while a worse response to VNS was related to the presence of vascular malformations and Lennox–Gastaut Syndrome.

**Discussion:**

We observed an association between a better response to VNS therapy no history of neurosurgery, focal interictal epileptiform activity, and focal seizure pattern. Additionally, it is important to highlight that age was not a determinant factor of the response, as children and adults had similar response rates. Thus, VNS therapy should be considered in both adults and children with DRE.

## Introduction

Epilepsy is a global health challenge, affecting an estimated 50 million individuals worldwide, with a considerable proportion inhabiting low- and middle-income nations (Borges et al., 2004; Singh and Sander, 2020). Despite the availability of antiseizure medications (ASMs), nearly a third of this population grapples with drug-resistant epilepsy (DRE), an inability to control seizures following adequate trials of two distinct ASMs (Kwan et al., 2010; Brodie et al., 2012; Pérez-Pérez et al., 2021). DRE presents not just a medical challenge but also introduces significant social, psychological, and economic complications (Wiebe et al., 1999).

While surgical intervention offers potential relief for some, many patients are either unsuitable candidates or do not experience the anticipated seizure control post-procedure. Consequently, exploring alternative treatments, such as dietary modifications like the ketogenic diet or neuromodulation techniques, becomes indispensable (Engel, 2014).

Among the various neuromodulation therapies, vagus nerve stimulation (VNS) has emerged as a promising adjuvant treatment for refractory epilepsy. VNS involves the implantation of a programmable generator in the subclavicular area that transmits electrical pulses to an electrode placed around the vagus nerve in the cervical region (Mertens et al., 2018). Although the precise therapeutic mechanisms of VNS remain elusive, emerging research indicates several potential factors influencing its efficacy.

In a comprehensive review by Englot et al. (2016), it was reported that approximately 8% of patients achieve seizure freedom within two to 4 years of VNS therapy, with nearly half to two-thirds experiencing at least a 50% reduction in seizure frequency. Other studies have recognized predictive factors of VNS treatment response, such as a more favorable outcome in patients with focal epilepsy, especially those without bilateral epileptic activity (Hachem et al., 2018). This correlation was initially demonstrated by Janszky et al. (2005) and later reaffirmed by Kim et al. (2017). Moreover, the pattern of interictal electroencephalograms (EEGs) among patients has been found to affect VNS efficacy (Ulate-Campos et al., 2015).

Intriguingly, the role of the autonomic nervous system in predicting VNS treatment outcomes has been a focus of recent research. Liu et al. (2018) have demonstrated how preoperative heart rate variations can predict the therapeutic response to VNS. In these studies, individuals with lesser impairment of parasympathetic cardiac control or greater vagal tone exhibited a higher likelihood of experiencing favorable responses to VNS treatment.

Furthermore, the utilization of computational models holds promise for enhancing the outcomes of neurostimulation in epilepsy. These models, which integrate biophysical principles, anatomical data, and patient-specific factors, can guide the optimization of stimulation parameters and personalization of treatment strategies (Dallmer-Zerbe et al., 2023). Nevertheless, the clinical application of these models remains an ongoing prospect rather than an established reality.

Despite more than two decades of VNS therapy in clinical practice, the identification of reliable predictors for treatment response continues to be a challenge (Hachem et al., 2018). It is crucial to ascertain the patients most likely to benefit from VNS therapy to guide patient selection and optimize treatment outcomes. Accordingly, this study aims to identify the indications, patient profiles, response patterns, and associated clinical characteristics among Brazilian patients from diverse regions of the country. By uncovering the factors influencing VNS treatment response, we seek to advance patient management, inform early intervention strategies, and ultimately enhance the overall effectiveness of VNS therapy in treating epilepsy.

## Methodology

### Study design and patients

This retrospective study reviewed data from patients with drug-resistant epilepsy (DRE) due to different etiologies who underwent VNS placement from 2005 to 2018, with a minimum follow-up of 6 months. Data were collected from the electronic databanks of six epilepsy centers in Brazil: Hospital Universitário Antônio Pedro, Universidade Federal Fluminense (HUAP-UFF), Rio de Janeiro; Instituto Estadual do Cérebro Paulo Niemeyer (IECPN), Rio de Janeiro; Instituto de Neurologia de Goiânia, Goiânia; Hospital das Clínicas da Universidade de São Paulo (HC-USP), São Paulo; Faculdade de Medicina do ABC, Santo André; Hospital de Clínicas – Universidade Federal do Paraná (UFPR), Porto Alegre; and Hospital Felício Rocho, Belo Horizonte. The study was approved by the Ethics Committee of Fluminense Federal University (IRB 14338019.0.0000.5243).

Patients included in the study were those who underwent VNS placement at any age, with no history of surgical treatment failure from 6 months to 13 years post-placement. Moreover, only those whose implanted devices were successfully activated without any complications during or after surgery, and had a minimum of 6 months of VNS therapy, were considered. All the patients underwent seizure recording in the epilepsy monitoring unit. The only exclusion criterion was having missing essential data. A total of 214 patients were selected from six epilepsy centers. Due to missing essential data, 22 patients were excluded, leaving 192 patients for analysis.

### Data collection and statistical analysis

Data collected included sex, age, presence of mental disability according to clinical standards set by the American Association on Intellectual and Developmental Disabilities, epilepsy etiology (unknown, genetic or structural-cortical development malformation, vascular malformation, tumor, encephalomalacia, infectious encephalitis), magnetic resonance imaging, electroencephalographic findings (generalized or focal and localization when possible), seizure classification according to the 2017 ILAE classification (Scheffer et al., 2017). Ictal recordings were obtained using prolonged video-electroencephalography before VNS implantation. All patients underwent surgical investigation with long term videoelectroencephalogram and seizure registration in a monitoring unit.

Response to treatment was defined as responders (≥ 50% reduction in debilitating seizures) and non-responders (< 50%) according to seizure frequency, as recorded in the patient’s chart. Response to treatment was also divided into the following categories according to the Engel modified classification: no response, response between 20 and 50%, between 50 and 90%, between 90 and 99, and 100% (seizure freedom).

Demographic and clinical data are described and expressed as means and standard deviations or median and range for continuous variables, as appropriate, and frequencies and percentages for categorical variables. Logistic regression models were used to estimate the probability of improvement (defined as a reduction of at least 50% of crises) according to each of these variables in the univariate and multivariate models. The analyses considered *p*-values <0.05 to be statistically significant and those between 0.06 and 0.10 to be of borderline significance. All analyses were conducted using IBM SPSS Statistics (version 21.0).

## Results

Included patients were aged 2–66 years (25.6 ± 14.3 years), 105 (54.7%) males, and 87 (45.3%) females. The median follow-up interval was 5 years (between 2005 and 2018). The included patients were on a median of 3 antiseizure medications (ASMs) (range, 2–5). Only 54 (31.2%) had normal results within the group with radiological exams available. In those with altered images (*n* = 118, 68.8%), we identified cortical malformation (*n* = 58, 43.9%), encephalomalacia (*n* = 48, 36.4%), vascular malformation (*n* = 1, 0.8%), and neoplasia (*n* = 1, 0.8%), and 10 patients presented with more than one type of alteration (*n* = 6, 5.1%) (Table 1).

**Table 1:** General characteristics of patients included in the study.

Variables	
N	192
Age	2–66 years (25.6 ± 14.3)
Median follow-up	5 years (range, 2005–2018)
Median ASMs	3 ASMs (range, 2–5)
	**Frequencies (%)**
Sex (female/male)	87/105 (45.3%/54.7%)
Intellectual disability	115 (64.6%)
Etiology	
Unknown	48 (25.0%)
Genetic	14 (7.4%)
Structural	120 (63.8%)
Multiple	6 (3.1%)
Abnormal MRI (*n* = 118)	118/172 (68.6%)
Cortical malformation	58 (43.9%)
Encephalomalacia	48 (36.4%)
Vascular malformation	1 (0.8%)
Neoplasia	1 (0.8%)
More than one type	10 (7.6%)
Abnormal electroencephalogram (EEG) (*n* = 184)*	178/184 (96.7%)
Focal interictal epileptiform discharges	117/178 (65.7%)
Generalized interictal epileptiform discharges	80/178 (44.9%)
Focal + generalized	18/178 (10.6%)
Seizure pattern (*n* = 190)	
Focal or multifocal seizure	99/190 (52.1%)
Generalized seizure	49/190 (25.8%)
Mixed seizure	45/190 (23.7%)
Neurosurgery	48 (25.0%)
Partial callosotomy	18/48 (37.5%)
Temporal lobectomy	13/48 (27.1%)
Focal temporal and extra temporal resections	10/48 (20.8%)
Association of callosotomy with other type of surgery	2/48 (4.2%)
Amygdalohippocampectomy	1/48 (2.1%)
Other types of surgery	4/48 (8.3%)

* Nine patients had missing electroencephalogram data.

**Figure 1:**
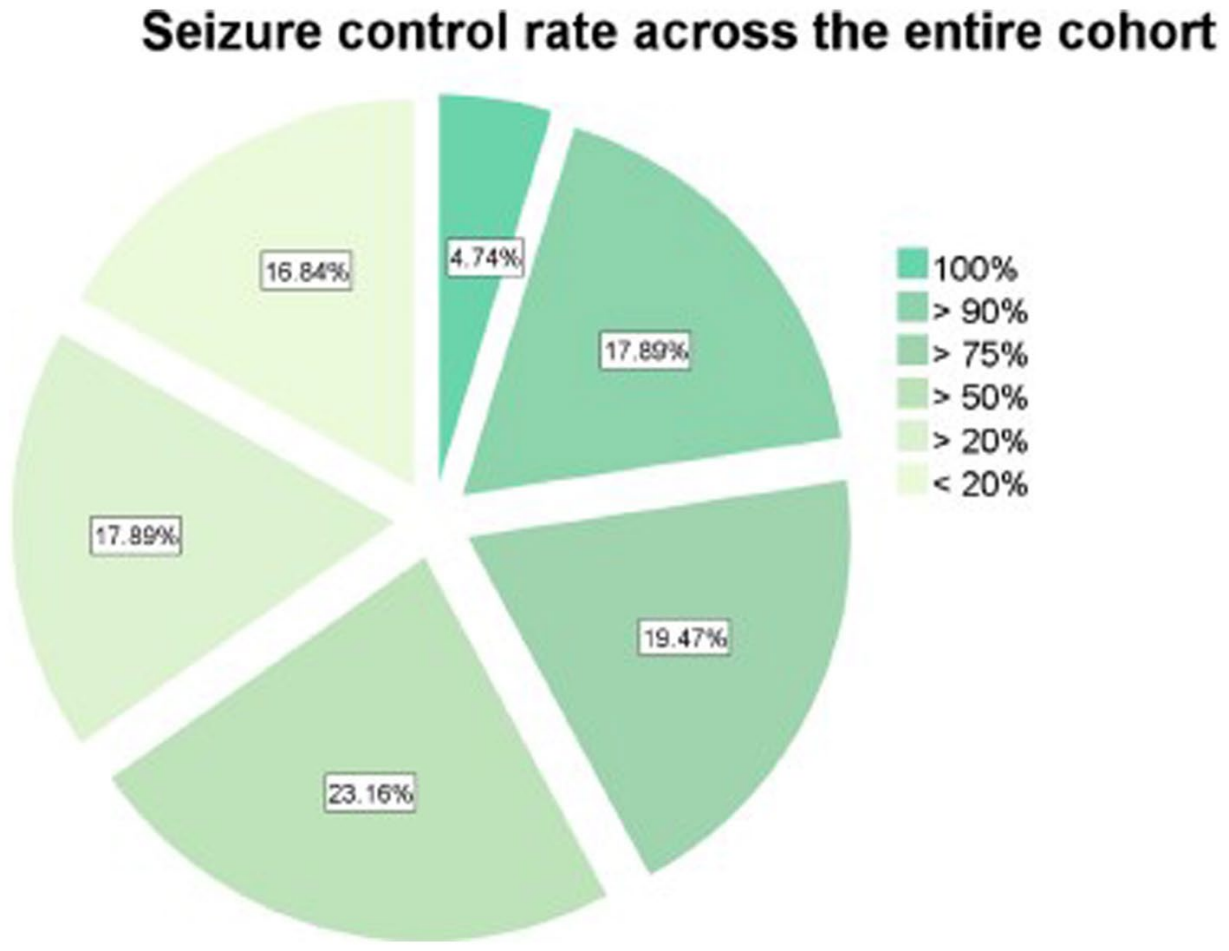
Seizure control rate achieved after vagus nerve stimulator placement across the entire cohort.

The seizure control outcomes were classified according to the modified Engel classification. Overall, the response rate (≥ 50% seizure reduction) after VNS implantation was 65.6% (126/192 patients). Most patients had a 50–90% seizure reduction (60.9%). Moreover, it is important to highlight that nine patients (4.7%) became seizure-free after VNS placement (Figure 1). There were no severe complications associated with VNS implantation, and the main adverse effects observed were mild to moderate cough (*n* = 114, 60%), hoarseness (*n* = 77, 40%), dyspnea (*n* = 39, 20%), and paresthesia (*n* = 39, 20%) in this cohort. There was no reported death among the patients. One patient had significant weight loss, but stabilized after the third month. No patient had to shut down the device due to adverse effects.

**Figure 2:**
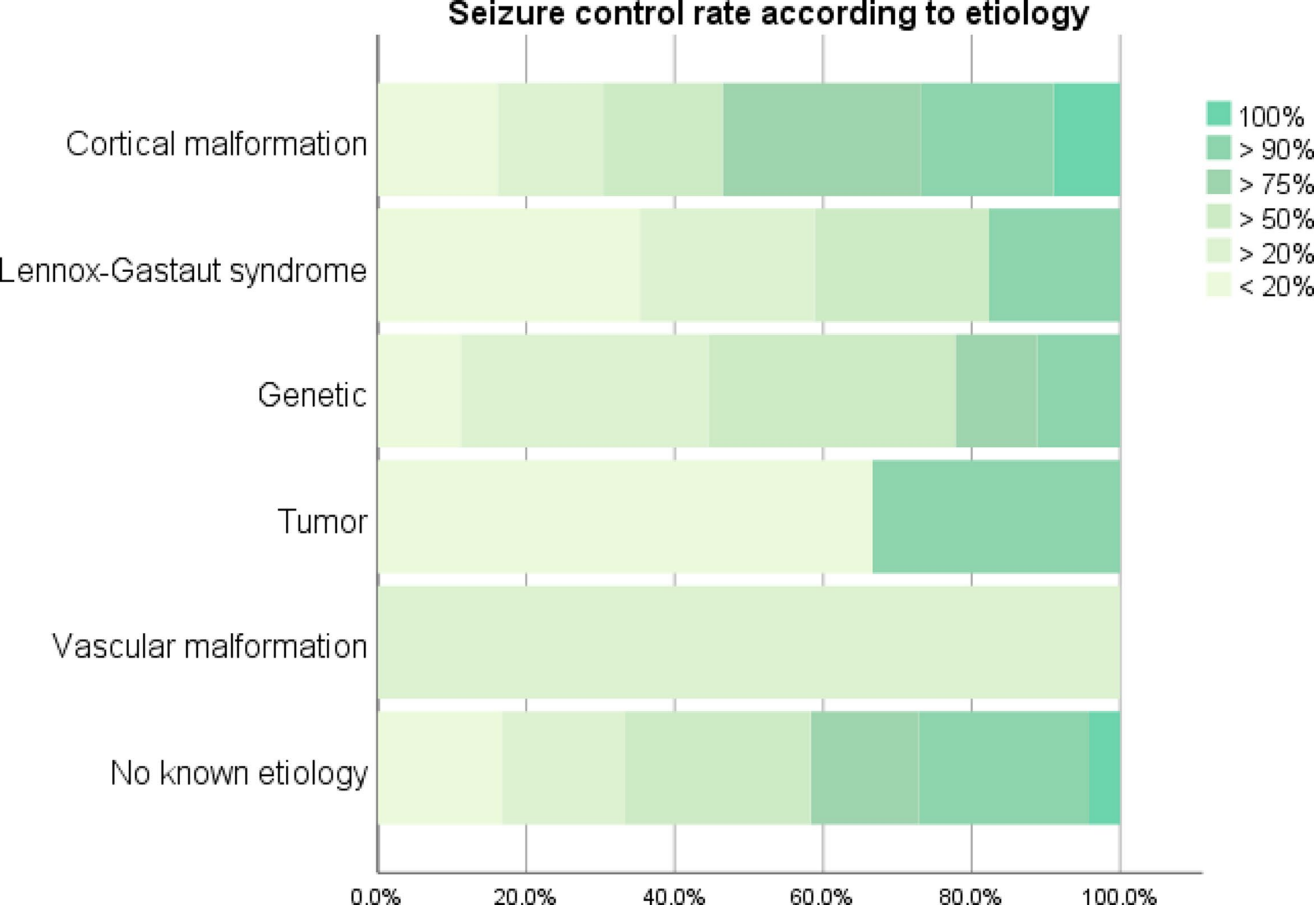
Seizure control rate achieved after vagus nerve stimulator placement according to etiology.

To predict which demographic and clinical characteristics of patients could be associated with the response to VNS implants, we compared the subgroups of patients according to response (≥ 50% seizure reduction, *n* = 126) or not (<50% seizure reduction, *n* = 126) or not (50% seizure reduction, *n* = 66). Although there was no difference in response when comparing children to adults or when comparing different etiologies (Figure 2), the rate of a ≥ 50% seizure reduction response was significantly higher in patients with no history of neurosurgery (*χ*^2^ = 6.763, *p* = 0.009). Patients with intellectual disability had lower odds of achieving a seizure rate of at least 50% (OR 0.46 [95% CI, 0.21–0.87], *p* = 0.029), but no difference in the odds of seizure freedom. There was no difference in seizure control associated with the presence of putative structural abnormalities, encephalitis, or encephalomalacia on the magnetic resonance image.

### Predictive value of epilepsy etiology

The predictive value of epilepsy etiology over seizure control rate after VNS placement was also investigated through an ordinal logistic regression model that included the presence of genetic syndromes, developmental cortical malformations, vascular malformations, intracranial neoplasms, and the diagnosis of Lennox–Gastaut Syndrome (LGS). Overall, etiological predictors of a better VNS response profile were tumors (*β* = 2.03, *p* = 0.002), while a worse response to VNS was related to the presence of vascular malformations (*β* = −1.261, *p* = 0.03) and LGS (*β* = −0.95, *p* = 0.024). Although age of VNS implant did not influence seizure control across the entire cohort, older age of VNS implantation was weakly related to poorer seizure control in patients with epilepsy related to genetic syndromes (*β* = −0.05, *p* = 0.003) and vascular malformations (*β* = −0.026, *p* = 0.006), but not to cortical malformations or LGS. Furthermore, there was no significant interaction between etiologies and neurosurgical treatment in terms of seizure control rate.

### Focal seizure and electrographic patterns predict greater seizure control

To address the influence of seizure type over the degree of seizure control after VNS implantation, we developed an ordinal logistic model that had as input the interaction between the presence of focal and generalized seizures. In this model, the presence of focal without generalized seizures was associated with a 1.58-fold (95% CI, 1.13–2.29) increase in the likelihood of achieving greater seizure control (*p* = 0.032). Similarly, patients with focal scalp EEG interictal patterns without generalized discharges achieved a 3.44 (95% CI, 1.50–9.50) times greater degree of seizure control (*p* = 0.006).

To further investigate the predictive value of scalp EEG discharge topography on seizure control response after VNS implantation in patients with focal EEG discharges, a similar ordinal logistic regression model was developed, estimating the main effects of focal interictal discharges and multifocal/generalized. Greater seizure control was seen in patients with focal discharges (OR 11.10 [95% CI, 4.50–7.3 × 10^9^], *p* = 0.001). In the entire cohort, similar results were found, revealing a better response in patients with focal spikes on interictal EEG (OR 12.91 [95% CI, 5.176–68.10], *p* = 0.002).

## Discussion

The findings of our retrospective multicenter study provide valuable insights into the factors influencing the response to VNS therapy in patients with refractory epilepsy. The median follow up was 5 years, and our results demonstrated a response rate of 65.6% for ≥50% seizure reduction relative to baseline, consistent with previous studies (Englot et al., 2016). Additionally, approximately 5% of patients achieved seizure freedom after VNS implantation, suggesting the potential of VNS as an effective treatment option for a subset of refractory epilepsy patients.

In line with previous literature, our study showed that individuals with focal epilepsy tend to respond better to VNS therapy compared to those with generalized epilepsy (Helmers et al., 2001; Rychlicki et al., 2006; Montavont et al., 2007; Ghaemi et al., 2010; Arcos et al., 2014; Kim et al., 2017). This observation aligns with the association we found between localized interictal and ictal epileptiform activity and a favorable VNS response, as supported by the findings of Arcos et al. (2014) and Kostov et al. (2022). However, the relationship between VNS responsiveness and other factors such as sex and age has not yet been definitively established and warrants further investigation (Labar, 2004; Janszky et al., 2005; Englot et al., 2017).

One important aspect we investigated was the potential variation in response rates between different age groups, specifically children and adults. Contrary to some previous studies that suggested differential response rates based on age (Ulate-Campos et al., 2015; Yazdi and Schumaker, 2016; Jain and Arya, 2021), our findings did not reveal a significant difference in VNS treatment response between children and adults in the entire population. This observation aligns with a growing body of literature that also reports similar response rates across age groups (Helmers et al., 2001; Orosz et al., 2014; Hilderink et al., 2017; Batson et al., 2022). It is noteworthy that our study cohort included patients from a diverse age range, allowing for a comprehensive analysis of VNS response across different developmental stages.

The lack of age-related differences in VNS treatment response is an important finding, as it suggests that VNS therapy can be equally effective and beneficial for both children and adults with refractory epilepsy. This has significant implications for clinical practice, as it supports the notion that age should not be a restricting factor when considering VNS as a treatment option. It is important to acknowledge that our findings are in contrast to some studies that have reported age-related variations in VNS response (Orosz et al., 2014; Ulate-Campos et al., 2015; Yazdi and Schumaker, 2016; Hilderink et al., 2017; Toffa et al., 2020; Batson et al., 2022). However, discrepancies in sample characteristics, study design, and methodology may contribute to these divergent results. The inclusion of a larger and more diverse patient population in our study may have enhanced the statistical power to detect any potential age-related differences in VNS response.

Further investigation is warranted to better understand the relationship between age and VNS treatment outcomes. Large-scale prospective studies with well-controlled designs are needed to provide more definitive evidence regarding the influence of age on VNS response in patients with refractory epilepsy. Additionally, exploring other factors such as etiology, seizure type, and comorbidities in relation to age could provide a more comprehensive understanding of the variables influencing VNS treatment outcomes.

Regarding etiological predictors, our study revealed varying responses based on the underlying cause of epilepsy. In line with the results of previous studies demonstrating that structural causes of symptomatic epilepsy responded better to VNS therapy (Colicchio et al., 2010; Touma et al., 2022), our results showed that patients with tumors had a better VNS response profile. Conversely, vascular malformations and Lennox–Gastaut syndrome (LGS) were associated with a poorer response to VNS. These results highlight the importance of considering etiological factors when evaluating the potential benefits of VNS treatment.

It was observed in this study that seizure reduction response was significantly higher in patients with no history of neurosurgery, which does not invalidate the indication of VNS therapy for this group as discussed in the study by Elliott et al. (2011).

Furthermore, in our results, the presence of intellectual impairment was associated with a decreased VNS response rate. Nonetheless, it is worth noting that VNS has been shown to positively influence general behavior and improve the quality of life of patients and their caregivers (Parker et al., 1999; Lagae et al., 2015). Therefore, non-clinical variables should also be taken into account when assessing the overall value of VNS therapy in patients with intellectual impairment.

It is important to acknowledge the limitations of our study. As a retrospective study, it is susceptible to observer bias. Furthermore, our analysis did not include an examination of quality of life and behavior data, nor did it explore the response rate and pattern in relation to the duration of epilepsy prior to VNS implantation. Finally, our work was not able to standardize the VNS parameters to analyze their relationship with the response in crisis control. Future studies should aim to address these limitations and investigate the impact of changes in specific subtypes of epileptic seizures, variations in anticonvulsant drug regimens, and modification protocols for VNS stimulation settings.

To better understand and more assertively predict VNS treatment responsiveness, future research should explore additional factors. Investigating connectivity patterns, including thalamocortical connections, has shown promise in predicting VNS response (Aldenkamp et al., 2002; Bartolomei et al., 2016; Ibrahim et al., 2017). Our findings align with previous studies indicating that a better response to VNS is associated with increased thalamocortical connectivity, particularly between the cingulate and insular cortices (Ibrahim et al., 2017; Yokoyama et al., 2020). Additionally, functional neuroimaging studies have revealed differences in thalamocortical circuitry and functional connectivity in patients with different types of epilepsy (Ibrahim et al., 2017; Yokoyama et al., 2020). Understanding these neural network dynamics could provide valuable insights into the mechanisms underlying VNS response and aid in refining patient selection criteria.

Moreover, investigating autonomic activity patterns and the involvement of the afferent vagal network in the VNS mechanism of action may provide further insights into VNS treatment responsiveness in individuals with focal seizures (Abela et al., 2014; Hachem et al., 2018; Hödl et al., 2021). Exploring the relationship between autonomic activity and VNS response could enhance our understanding of the underlying mechanisms and potentially contribute to improved patient selection.

The present study does not address whether the sidedness of focal epilepsy (left vs. right) has any impact on the response to VNS therapy. Given that the maladaptive epileptogenic networks tend to strengthen on the side of the putative lesion, and by also acknowledging that the autonomic brainstem involvement with cortical and subcortical networks has a bilateral (rather than sided) pattern (Hildebrandt et al., 2021), we can speculate that responsivity to VNS would be similar in left- and right-sided focal epilepsy cases. However, due to a lack of data on the focal epilepsy sidedness of the included participants, we cannot address this important question at this time. This would be a valuable avenue for further investigation in future studies, as these insights could potentially help in the further refinement of patient selection criteria for VNS therapy and personalizing treatment plans for better outcomes in patients with drug-resistant epilepsy. Thus, while this study sheds light on major VNS response predictors, the role of epileptic focus sidedness in VNS therapy needs further study.

Our retrospective multicenter study provides crucial insights into the factors influencing VNS response in patients with refractory epilepsy. The findings support the efficacy of VNS therapy in reducing seizure frequency, particularly in patients with focal epilepsy and certain etiologies such as tumors. Importantly, our study demonstrates that VNS treatment is equally effective for both children and adults, challenging the notion of age as a restricting factor. Future research should focus on exploring connectivity patterns and autonomic activity to refine patient selection criteria and optimize the prediction of VNS treatment responsiveness, ultimately improving outcomes for individuals with drug-resistant epilepsy. By identifying these predictive factors, we can better personalize VNS therapy and enhance its clinical applicability in the management of refractory epilepsy.

## Data availability statement

The original contributions presented in the study are included in the article/supplementary material, further inquiries can be directed to the corresponding author.

## Author contributions

ID’A-M, LFP, DB, LP, FA, and AO contributed to conception and design of the study. HP organized the database. JS performed the statistical analysis. HP, TR, and ID’A-M wrote the first draft of the manuscript. ID’A-M, JS, and VL wrote sections of the manuscript. All authors contributed to manuscript revision, read, and approved the submitted version.

## Conflict of interest

The authors declare that the research was conducted in the absence of any commercial or financial relationships that could be construed as a potential conflict of interest.

## Publisher’s note

All claims expressed in this article are solely those of the authors and do not necessarily represent those of their affiliated organizations, or those of the publisher, the editors and the reviewers. Any product that may be evaluated in this article, or claim that may be made by its manufacturer, is not guaranteed or endorsed by the publisher.
